# Binge Drinking of Ethanol during Adolescence Induces Oxidative Damage and Morphological Changes in Salivary Glands of Female Rats

**DOI:** 10.1155/2016/7323627

**Published:** 2016-08-07

**Authors:** Nathalia Carolina Fernandes Fagundes, Luanna Melo Pereira Fernandes, Ricardo Sousa de Oliveira Paraense, Paulo Mecenas Alves de Farias-Junior, Francisco Bruno Teixeira, Sergio Melo Alves-Junior, João de Jesus Viana Pinheiro, Maria Elena Crespo-López, Cristiane Socorro Ferraz Maia, Rafael Rodrigues Lima

**Affiliations:** ^1^Laboratory of Functional and Structural Biology, Institute of Biological Sciences, Federal University of Pará, Street Augusto Corrêa 1, Guamá, 66075-900 Belém, PA, Brazil; ^2^Laboratory of Inflammation and Behaviour Pharmacology, Pharmacy Faculty, Institute of Health Science, Federal University of Pará, Street Augusto Corrêa 1, Guamá, 66075-900 Belém, PA, Brazil; ^3^Laboratory of Molecular Pharmacology, Institute of Biological Sciences, Federal University of Pará, Street Augusto Corrêa 1, Guamá, 66075-900 Belém, PA, Brazil; ^4^Department of Oral Pathology, School of Dentistry, Federal University of Pará, Street Augusto Corrêa 1, Guamá, 66075-900 Belém, PA, Brazil

## Abstract

This study investigates morphological and biochemistry effects of binge ethanol consumption in parotid (PG) and submandibular (SG) salivary glands of rats from adolescence to adulthood. Female Wistar rats (*n* = 26) received ethanol at 3 g/kg/day (20% w/v) for 3 consecutive days/week from the 35th until the 62nd day of life. Animals were treated in two periods: 1 week (G1) and 4 weeks (G2), with a control (treated with distilled water) and an ethanol group to each period. In morphological analysis, morphometric and immunohistochemistry evaluation for smooth muscle actin (*α*SMA), cytokeratin-18 (CK-18), and vimentin (VIM) were made. Biochemical changes were analyzed by concentration of nitrites and levels of malondialdehyde (MDA). The difference between groups in each analysis was evaluated by Mann-Whitney* U* test or Student's *t*-test (*p* ≤ 0.05). PG showed, at one week of ethanol exposure, lower CK-18 and *α*-SMA expression, as well as MDA levels. After four weeks, lower CK-18 and higher MDA levels were observed in PG exposed to ethanol, in comparison to control group. SG showed lower *α*-SMA expression after 1 and 4 weeks of ethanol exposure as well as higher MDA levels after 1 week. Ethanol binge consumption during adolescence promotes tissue and biochemical changes with only one-week binge in acinar and myoepithelial PG cells.

## 1. Introduction

According to World Health Organization (WHO), individuals over 15 years of age consume on average 6.2 liters of pure alcohol per year, which translates into 13.5 g of pure alcohol a day. America and Europe are regions shown as consuming above average. In 2012, 5.9% of global mortality was associated with alcohol use [1].

In this context, there are an increased potential and intense consumption of ethanol (EtOH) in women, especially in regions such as the United States [2], Brazil [3], and western Europe [4, 5]. This type of alcohol intake is often seen in younger women. Starting, in many cases, during adolescence, the easy access and lack of legal consequences may categorize this substance as one of the most commonly used drugs within this age group [6].

Heavy alcohol consumption, with high doses of EtOH for a short period of time followed by a period of abstinence, in a binge style, has a high prevalence and increases in frequency throughout adolescence, peaking in young adults and subsequently declining with advancing age [7–9].

EtOH consumption has been associated with triggering damage to organs and body tissues, such as the upper gastric tract, skeletal muscles, and salivary glands, which can cause morphological and functional changes [10, 11].

Salivary glands and their resulting secretion have a huge importance in the maintenance of oral and general homeostasis [12]. The structure of these glands comprises a series of ramified ducts that culminate in a secretory terminal portion, the acini cells, which compose the glandular parenchyma [12, 13]. However, the effects of usual consumption of EtOH intermittent in a binge drinking pattern on salivary glands have not been elucidated yet.

The investigation of changes in salivary glands after alcohol intake may involve analysis of immunohistochemical markers in parenchyma and stroma, including cytokeratin, vimentin, and alpha smooth muscle actin, intended to evaluate morphological alterations [13]. Biochemical analysis, as revealed by oxidative stress, also shows changes that can occur in pathological situations, as in the case of EtOH chronic consumption, showing involvement of oxidative stress in parotid gland sialadenosis [14].

This study aimed to evaluate the effects caused by episodic and intense intoxication of EtOH in a 3-day/week binge pattern on the parotid and submandibular salivary glands of female rats, during adolescence to young adulthood phase.

## 2. Materials and Methods

### 2.1. Animals and Experimental Groups

Female Wistar rats, 35 days old, were kept in standard conditions of temperature, in a climate-controlled room on a reverse light/dark cycle of 12 hours (lights on 7:00 AM), with food and water* ad libitum*. This study was approved by the Ethics Committee on Experimental Animals of the Federal University of Pará (CEPAE-UFPA: 196-14) and followed the guidelines suggested by the NIH* Guide for the Care and Use of Laboratory Animals*, with all animals kept in collective cages (maximum of five animals per cage). Female rats were chosen due to alcohol intake profile exhibited during adolescence, which is similar between males and females, representing the period of life in which women drink much more [15].

A sample size calculation was performed assuming a normal distribution of the variables tested. A power of 80% and a bilateral alpha level of 5% were assumed with standard deviation of 1.29 (ethanol group) and 0.20 (control group). Standard deviation was determined through a previous study [13]. Thus, a sample size of eight animals in ethanol group and five animals in control group (*n* = 26) was established for this study.

All animals received EtOH at a dose of 3 g/kg/day (20% w/v), simulating a pattern of binge consumption previously described [16], or distilled water, administered through gavage with orogastric cannula, for three consecutive days/week in the animals, 35 to 62 days old, which corresponds to late adolescence and early adulthood in this animal model [17, 18]. The weighing of the animals was performed weekly for dose adjustment.

**Figure 1:**
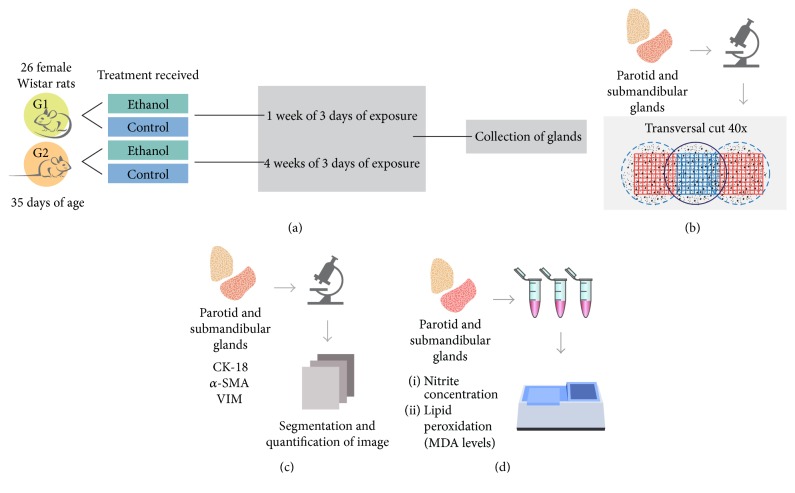
Sample description and experimental steps. Animals and sample description (a); morphometric analysis steps (b); immunohistochemistry analysis steps (c); oxidative stress assays (d).

The total sample was divided into two groups, according to the period of solution administration: G1, with 1 week of 3 days of exposure to ethanol/distilled water; and G2, with 4 weeks of 3 days of exposure to ethanol/distilled water. Each group was composed of a control group, where the animals received distilled water, and an ethanol group, where animals received EtOH. Twelve hours after treatment, animals were divided and submitted to collection of fresh glands or perfusion (*n* = 5–8 animals per group, Figure 1(a)).

### 2.2. Blood Alcohol Concentration (BAC)

Blood Alcohol Concentration (BAC) was obtained on postnatal day 37, after 60 minutes of alcohol intake, in the last three days of the binge drinking model exposure. Blood samples (*n* = 5–8 animals per group) were collected by intracardiac pathway, transferred to heparinized tubes, and then stored at 4°C until the EtOH concentration was measured. Briefly, homogenized blood sample (500 *μ*L) was added to butanol as internal standard (500 *μ*L) and analyzed by the head space gas chromatography (GC/MS) with Flame Ionization Detector (FID) on GC-MS Varian equipment (Walnut Creek, CA, USA), model CP3800, equipped with automatic injection of samples (CombPalm Series number 1210469). The capillary column (CP WAX 52 CB, Varian) was used, consisting of polyethylene (100%), with dimensions of 30 m × 0.32 mm × 0.25 *μ*m. The temperature of the detector was 250°C and injector was 200°C.

### 2.3. Euthanasia of Animals and Collection of the Glands

The animals were anesthetized with a combination of ketamine hydrochloride (90 mg/kg) and xylazine (10 mg/kg). After the absence of corneal and paw withdrawal reflex, surgery was performed to collect the parotid and submandibular salivary glands. The gland of the right side was removed and used for oxidative stress analysis. The gland of the left side was removed after perfusion and used to immunohistochemistry and morphometric analysis.

All glands were weighed after removal by analytical balance (FA 2104 N, Electronic Balance Bioprecisa, Shanghai, China), and the relative glandular weight was calculated (gland weight × 100/final body weight).

### 2.4. Immunohistochemistry and Morphometric Analysis

After perfusion of the animals, one submandibular and one parotid gland from the left side of each animal were postfixed in 6% formaldehyde until processing [19, 20]. The glands were dehydrated in increasing ethanol battery, cleared in xylene, and embedded in Paraplast resin, available for further 3 *μ*m sections.

With regard to morphometric assays, the mean percentage equivalent to the glandular parenchyma and stroma region was evaluated, with evaluation of two slides per animal: one submandibular and one parotid gland. The area of the samples was evaluated by the planimetry method for counting points, using ImageJ software version 1.33-1.34 (NIMH, NIH, Bethesda, MD, USA, http://rsbweb.nih.gov/ij/). All sections were stained in hematoxylin and eosin, with five aleatory images of each gland, captured in 40x magnification on Axio Scope microscope (Carl Zeiss, Germany) equipped with a CCD colour camera AxiocCam HRC (Carl Zeiss, Germany). After this step, a grid with equidistant points was applied in each image (NIMH, NIH, Bethesda, MD, USA, http://rsbweb.nih.gov/ij/). The coincident points with glandular parenchymal and stromal elements were counted, and the percentage of each region was calculated by dividing the number of incidents on the same points and the total number of points on the grid, separately [13] (Figure 1(b)).

Immunohistochemical studies were performed on paraffin-embedded tissues using the streptavidin (Reveal Spring, Pleasanton, CA, USA) and 3,3-diaminobenzidine (DAB; Sigma, USA) methods. Briefly, 3 *μ*m sections were deparaffinized and rehydrated in battery with decreasing concentration of alcohol. After antigen retrieval chamber Pascal (Dako Corporation, Carpinteria, CA, USA) and blocking of endogenous peroxidase activity, the sections were incubated in primary antibody anti-*α* smooth muscle actin (*α*-SMA) (1 : 50, Dako), anticytokeratin-18 (CK-18) (1 : 100 Bioss), and antivimentin (VIM) (1 : 100, Bioss). Subsequently, the sections were incubated for 30 min with biotin-free horseradish peroxidase (HRP) enzyme-labeled polymer (REVEAL, Spring, Pleasanton, CA, USA). Diaminobenzidine (Sigma Chemical Corp., St. Louis, USA) was used as the chromagen and the sections were counterstained with Mayer's hematoxylin (Sigma Chemical Corp., St. Louis, USA).

Immunohistochemical analysis of two slides per animal was performed, one submandibular and one parotid gland. The cytoplasmatic expression of the proteins studied was measured throughout the glandular parenchyma, by evaluation of the extent of the area (*μ*m) and fraction (%) of a marked section (brown stain intensity). Brightfield images of five randomly selected areas for each sample were acquired with Axio Scope microscope (Carl Zeiss, Germany), equipped with a CCD colour camera AxiocCam HRC (Carl Zeiss, Germany), with a magnification of 40x. Areas stained by DAB were separated and segmented using “colour deconvolution plug-in” (Gabriel Landini, http://www.dentistry.bham.ac.uk/landinig/software/software.html) from ImageJ software version 1.33-1.34 (NIMH, NIH, Bethesda, MD, USA, http://rsbweb.nih.gov/ij/). After image segmentation, the area and fraction of total staining were measured (Figure 1(c)).

### 2.5. Oxidative Stress Assays

After collection and weighing of glands, the extracted tissue was rinsed in saline and subjected to freezing in liquid nitrogen and subsequently stored at −80°C. For analysis, samples were thawed and resuspended in 20 mM Tris-HCl buffer, pH 7.4, at 4°C for sonic disintegration (approximate concentration of 1 g/mL) (Figure 1(d)).

The concentration of nitrite was determined based on a reaction with Griess reagent (0.1% naphthyl-ethylene-diamine and 1% sulfanilamide in 5% phosphoric acid; 1 : 1). An aliquot of crude homogenate was centrifuged at 21,000 g for 20 min at 4°C, and the supernatant was used to analyze nitrite levels such as described elsewhere [21].

Briefly, fifty microliters of the supernatant or standard nitrite solution was added to 50 mL of Griess reagent and incubated for 20 minutes at room temperature. The absorbance was measured at 550 *η*m and compared to that of standard solutions of sodium nitrite.

The level of lipid peroxidation was determined by the method proposed by Esterbauer and Cheeseman, based on measurement of malonaldehyde (MDA) and 4-hydroxyalkenals (4-HA) levels [22]. Briefly, an aliquot of crude homogenate was centrifuged at 2,500 g for 30 min at 4°C, and supernatant was processed as described by the Bioxytech LPO-568 kit (Cayman Chemical). This kit takes advantage of a chromogenic reagent that reacts with MDA and 4-HDA at 45°C, yielding a stable chromophore with maximal absorbance at the 586 *η*m wavelength.

Quantities of total protein content in the supernatants (used for determination of lipid peroxidation and nitrite levels) were assayed as described previously [23]. Thus, after correcting for protein concentration, results of lipid peroxidation and nitrite levels were expressed in picomole per milligram of protein.

### 2.6. Statistical Analysis

The mean values were obtained from the control and EtOH groups, among parotid and submandibular glands, in each period evaluated, for each morphologic and biochemical assay. The normality of the data was verified by the Shapiro-Wilk test. The Student *t*-test was applied for normal data and the Mann-Whitney* U* test for abnormal data. All analyses were tabulated in GraphPad Prism software version 1.5 (San Diego, CA, USA). Statistical significance values of *p* < 0.05 were considered acceptable.

## 3. Results

### 3.1. BAC, Body Weight, and Relative Glandular Weight

**Figure 2:**
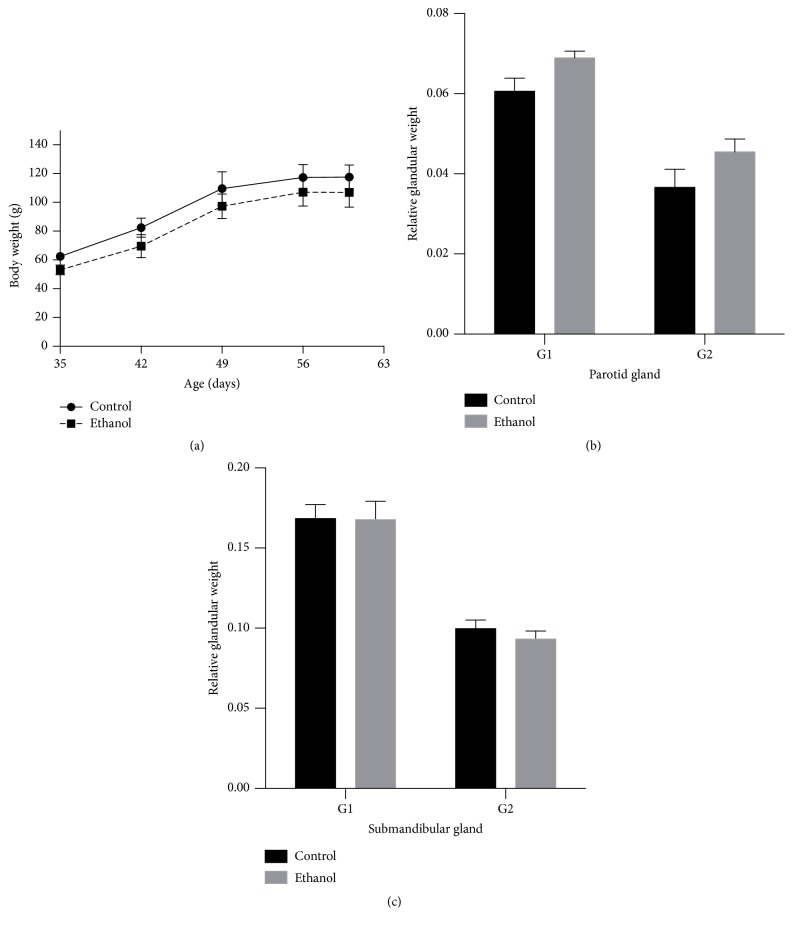
Effects of binge drinking (EtOH administration) during adolescence in the body weight gain of rats (a) and relative gland weight of parotid (b) and submandibular (c) glands. The results were expressed as mean ± SEM after Student's *t*-test.

The measurement of BAC 1 hour after the last three binge drinking exposures reached 197.4 ± 19.46 mg/dL. Besides, in both periods of treatment (35 to 41 days of age and 35 to 62 days of age), the ethanol intake did not alter the body weight of animals (Figure 2). No death was observed after our alcohol paradigm. In the analysis of relative glandular weight and the treatment received, no difference was detected in submandibular or parotid gland for both periods of analysis (Figure 2).

### 3.2. Morphologic Changes

**Figure 3:**
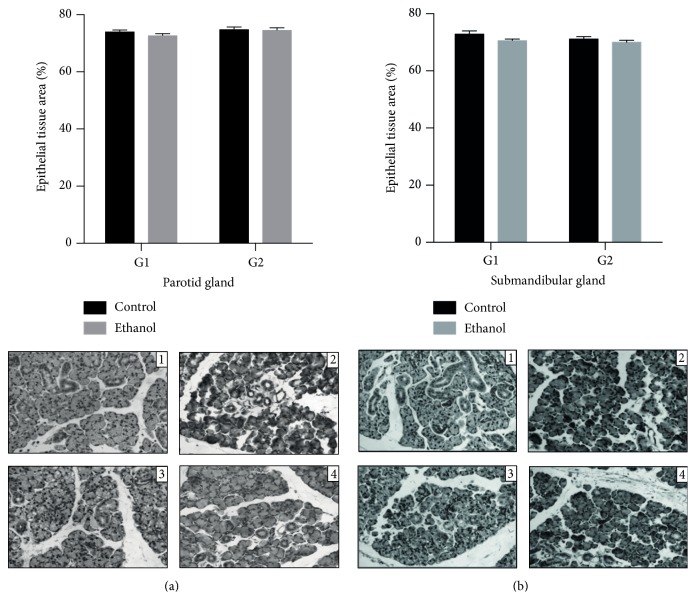
Morphometric analysis: effects of binge drinking (EtOH administration) during adolescence and young adulthood. Parotid (a) and submandibular (b) glands, after one week (control: 1, ethanol: 2) and four weeks of ethanol exposure (control: 3, ethanol: 4), according to treatment group. These results were expressed with photomicrographs and mean ± SEM (Mann-Whitney* U* test). Inset scale = 20 *μ*m.

In the morphometric assay, as the results show in Figure 3, no changes were observed in the total epithelial area to parotid and submandibular glands after exposure to ethanol in doses of 3 g/kg/day (Figure 3).

**Figure 4:**
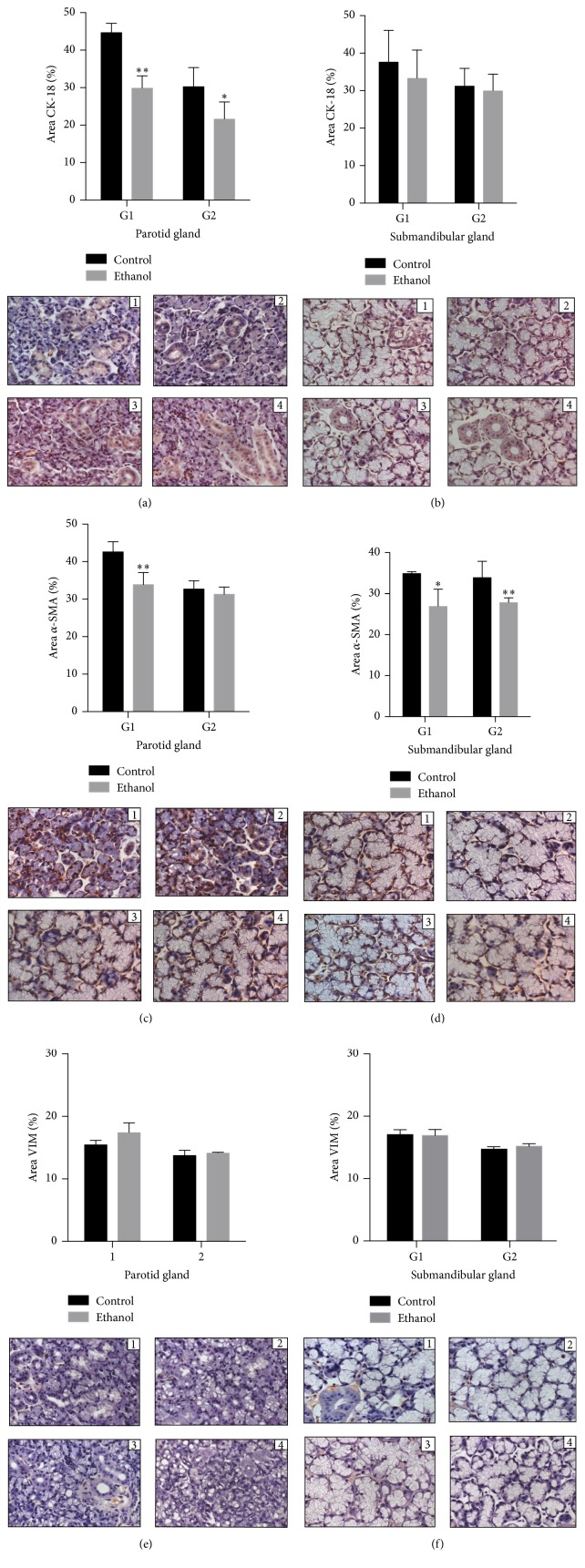
Effects of binge drinking (EtOH administration) during adolescence on the parotid and submandibular gland immune expressions. Anti-*α* muscle actin (*α*-SMA), anticytokeratin-18 (CK-18), and vimentin (VIM) positive cells of female Wistar rats after 1-week and 4-week exposure to ethanol in control group (1 and 3, at 1 week and 4 weeks, in each group) and ethanol (2 and 4, at 1 week and 4 weeks, in each group). The results were expressed as mean ± SEM. ^*∗*^ *p* ≤ 0.05 compared to control group (Mann-Whitney* U* test), ^*∗∗*^ *p* ≤ 0.01 compared to control group (Mann-Whitney* U* test).

In parotid glands, ethanol consumption affected expression of CK-18 at one week and four weeks of exposure (Figure 4(a)). When we analyzed the *α*-SMA, the difference among groups was only detected at one-week exposure to ethanol (Figure 4(c)). With regard to vimentin expression, no difference was detected in any of the periods of exposure (Figure 4(e)).

On the other hand, submandibular gland showed no difference between the control and ethanol groups in both periods of analysis regarding the expression of CK-18 (Figure 4(b)). The same was observed for vimentin expression (Figure 4(f)). As for expression of *α*-SMA, a lower expression was observed in ethanol group after one and four weeks of exposure (Figure 4(d)).

### 3.3. Oxidative Stress

**Figure 5:**
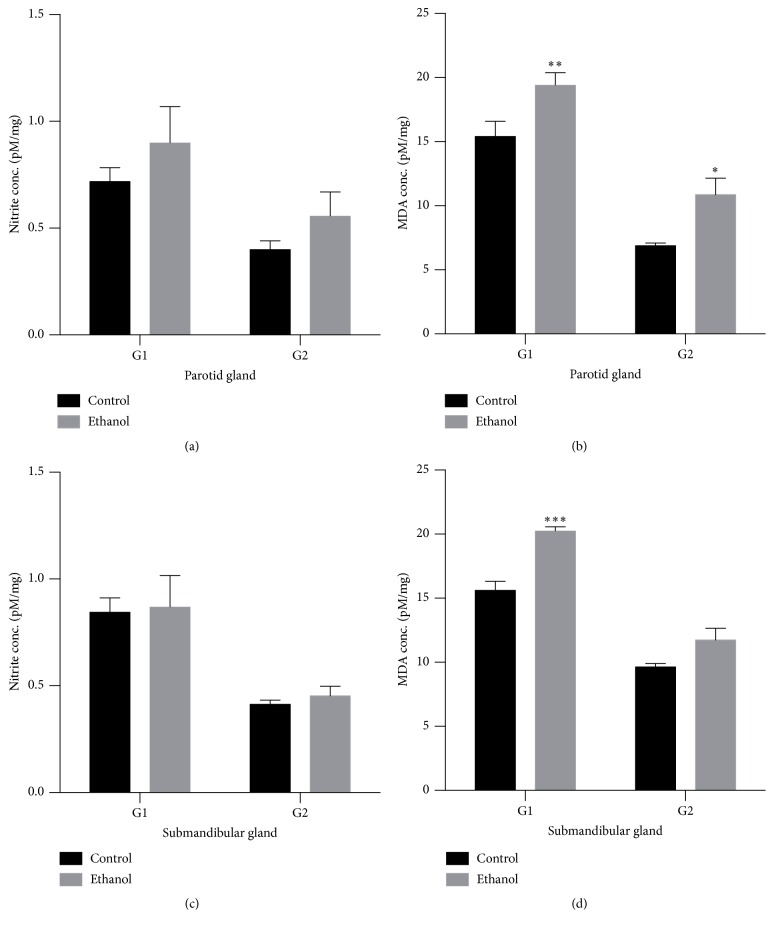
Effects of binge drinking (EtOH administration) during adolescence and young adulthood on the parotid and submandibular glands. The results were expressed as means ± SEM of the nitrite concentration (pM) per milligram of protein after 1 week (G1) and 4 weeks of ethanol exposure (G2) on parotid (a) and submandibular (c) glands and malondialdehyde (MDA) concentration (in pM) per milligram of protein after 1 week (G1) and 4 weeks of ethanol exposure (G2) on parotid (b) and submandibular (d) glands. ^*∗*^ *p* ≤ 0.05 compared to control group (Student's *t*-test), ^*∗∗*^ *p* ≤ 0.01 compared to control group (Mann-Whitney* U* test), and ^*∗∗∗*^ *p* ≤ 0.001 compared to control group (Mann-Whitney* U* test).

No difference in nitrites concentration among the control and ethanol groups was detected in parotid glands and submandibular glands (Figures 5(a) and 5(c)) for both periods of evaluation.

The levels of MDA showed an increase in expression in parotid glands at one and four weeks of exposure to ethanol (Figure 5(b)). Submandibular glands showed a higher level of MDA in ethanol group only at one week of exposure to ethanol (Figure 5(d)).

## 4. Discussion

In this study, the effects of an episodic binge drinking model of consumption of ethanol in salivary glands were investigated in female rats considering two different periods, one and four weeks of exposure. Our results showed, for the first time, that a single episode of ethanol binge drinking can affect the parenchyma of the parotid gland, reduce myoepithelial cells, and increase the levels of MDA in the parotid and submandibular glands. Furthermore, a 4-week exposure in the same conditions reveals a reduction in cytokeratin expression and MDA levels of the parotid gland and a reduction of the myoepithelial cells in the submandibular gland.

Our group have studied the effects of alcohol on the salivary glands of female rats from adolescence until adulthood. Our first findings demonstrated that a chronic heavy ethanol paradigm (6.5 g/kg/day) induces morphologic changes in both glands, however, in a different way. In parotid glands, an increase in total gland weight was observed as well as atrophy of glandular parenchyma. On the other hand, heavy ethanol intake promoted an increase in the submandibular gland stroma area. In addition, our work demonstrated through an immunohistochemistry assay that alcohol exposure increased duct-like cells related to caspase-3 overexpression in submandibular glands. These results highlight the difference between parotid glands and submandibular glands in the face of chemical noxious stimuli [13].

After that, we decided to investigate whether habitual and recreational consumption of alcohol among adolescents promotes the same range of damage in the salivary glands. Therefore, we employed the current protocol in doses that mimic a binge drinking pattern (3 g/kg/day for three days a week) [16]. According to the National Institute on Alcohol Abuse and Alcoholism (NIAAA), binge drinking is defined as a pattern of drinking that brings BAC levels of 0.80 mg/dL [24]. Our alcohol protocol reached BAC levels of 190 mg/dL, which is defined as heavy drinking (five or more drinks on the same occasion on ≥5 days in the last month and BAC starting at 80 mg/dL), that consists of higher prevalence rates among adolescents [15].

The evaluation of two different periods of exposures to EtOH shows the effects of an acute (1 week) and chronic (4 weeks) heavy binge drinking model in salivary glands [16]. In this study, a 1-week and 4 week binge result in similar responses at morphologic and biochemical evaluations, although a pattern breakage was observed in *α*-SMA expression in parotid gland and in MDA levels of submandibular glands. In both cases, only an acute exposure results in changes of these glands, suggesting an adaptation to damage in a period of chronic binging.

In this study, the ethanol group showed conformity of response with the chronic heavy drinking model in the expression of cytokeratin and *α*-SMA [13], like the MDA levels [14] reported in previous studies. Binge consumption of ethanol, especially in adolescence and early adulthood, has been associated with alcoholism with the hypothesis that binge drinking may indicate a qualitatively similar imbalance but quantitatively lower when compared with a heavy chronic drinking model [25].

In contrast, neither period of ethanol exposure was able to cause changes in the EtOH group when compared to the control group or modify parotid or submandibular glands in terms of size or parenchyma volume, although it has been shown that heavy chronic consumption of ethanol can increase the size and cause atrophy of the parenchyma area of the parotid gland [13, 14]. This lack of alterations may be associated with the dosage of consumption of ethanol observed in a heavy model, but further studies are necessary to clarify this process.

In line with a heavy chronic model of exposure to EtOH, morphologic changes after binge drinking are more related to expression patterns of CK-18 and *α*-SMA. CK-18 is commonly detected in the cytoskeleton of serous acinar and ductal cells in salivary glands. This protein helps to maintain cellular integrity [26, 27].

Parotid glands showed a reduction in CK-18 expression in 1- and 4-week episodic binges, while submandibular glands did not present any significant modification. A previous study exhibits a reduction of cytokeratin expression in parotid glands and an increase in submandibular glands after chronic ethanol exposure [13]. These data are related to what was observed in our investigation but showed that one episodic binge can change parotid gland parenchyma but is not capable of interfering with submandibular expression, suggesting the possibility of induction of a cytoprotection process in the parenchyma of these glands.

The changes observed in *α*-SMA, with a lower expression in parotid glands after one episodic ethanol binge or after 1 and 4 weeks of binge drinking in submandibular glands, are directly related to myoepithelial cells. These structures are associated with the propagation of neural stimuli, tumor suppression, and contraction and transportation of metabolites [28]. A reduction of the myoepithelial cell population has been associated with ductal atrophy [29].

Intended to analyze stromal fibroblasts, vimentin expression was also verified in this study and no difference among groups was detected in either the periods or glands evaluated. Vimentin symbolizes a mesenchymal marker of cell migration and invasion identifying activated fibroblasts or the myofibroblasts in salivary glands [30]. Such evidence shows conformity with morphometric findings and suggests that morphological changes after episodic binges are associated with changes in the parenchyma of the evaluated glands.

Alcohol consumption is also connected with a metabolic imbalance of free radical production, leading to oxidative stress. In this study, a higher level of MDA, a lipid peroxidation marker, was demonstrated in the parotid glands of the ethanol group after 1 and 4 weeks of ethanol intake in a binge model. Submandibular glands exhibited a higher level of lipid peroxidation in the ethanol group only after a 1-week binge treatment. No changes between groups were detected in nitric oxide analysis for both glands in 1-week or 4-week binge ethanol exposure. Previously, it was shown that increased levels of lipid peroxidation, detected by the production of toxic compounds such as MDA, were related to membrane damage and can be deleterious for membrane permeability [14, 31]. These toxic compounds can also initiate the production of abnormal substances, such as DNA and RNA [32].

A difference in response between parotid and submandibular glands was also shown by Fernandes et al. [13]. This study reported a difference in the caspase-2 expression pattern of response after heavy drinking consumption, in which only submandibular glands showed a greater expression of caspase-2, indicating greater levels of apoptosis in this tissue.

In the present research, differences between parotid and submandibular responses were related. As we know, these glands have metabolic and structural differences. Parotid glands present purely serous acini and submandibular glands show mucous acini carrying a terminal cap of serous cells [33, 34]. It was reported that parotid glands present essentially aerobic metabolism, while the metabolism of submandibular glands is primarily anaerobic [34]. This contrast between glands may be connected to the different regulatory mechanisms of enzymatic release reported by Busch and colleagues [35].

## 5. Conclusions

For the first time, in this study, 1 week of episodic binge drinking in female rats has been connected with damage to the submandibular and parotid glands showing a reduction in myoepithelial cells, cytokeratin expression in parenchyma, and higher levels of lipid peroxidation. On the other hand, different patterns of response were observed after 1-week and 4-week exposure to ethanol. Further studies are necessary to clarify the damage with longer periods of exposure to ethanol.
